# Health as a Multispecies Phenomenon: Towards Anti-Anthropocentrism

**DOI:** 10.1177/27539687261452260

**Published:** 2026-05-27

**Authors:** Guillem Rubio-Ramon, Krithika Srinivasan

**Affiliations:** 1 School of Geosciences, 70448 University of Edinburgh, UK

**Keywords:** multispecies health, anthtropocentrism, onto-epistemology, more-than-human geography, multispecies justice

## Abstract

This article develops a critical review of how geography and cognate social sciences have engaged with health as a ‘multispecies’ or ‘more-than-human’ phenomenon in the context of nonhuman animals. We find that these literatures have employed some key ontological and epistemological innovations to address the human-centredness of dominant health approaches, including those, such as Planetary Health, that strive to integrate human, animal, and ecological health. In doing so, they have been successful in advancing fuller understandings of the multispecies dimensions of health. However, our analysis suggests that they have had limited results in rethinking health in ways that are equitable across species. Building on this review, the article draws on multispecies justice scholarship to identify three conceptual avenues that offer promise in terms of generating anti-anthropocentric accounts of multispecies health: in-difference, intersubjectively affirmed agency, and re-animalisation. Attentive to how animal health and ill-health are deeply embedded within human systems, we conclude by examining how these three approaches might provide us with the necessary ‘reflective’ tools for more equitable accounts of multispecies health.

## Introduction

Health is and has always been more-than-human. Humans experience health through their (multispecies) bodies and in connection to the multispecies environments that they inhabit. Equally, the nonhuman lifeforms that constitute these multispecies worlds experience health through their (multispecies) bodies and in connection to their multispecies environments, which include humans. Yet, ideas and practices of health have mostly remained centred on humans, and to a lesser extent, on some valued nonhuman beings (nonhumans for short).

A range of geographical and critical social science literatures have conceptualised and empirically investigated the multispecies or more-than-human dimensions of health, with nonhuman animals as a prominent focus. A core concern has been implications for human health and wellbeing (e.g., Kirk et al., 2019; Wells, 2009), for instance, in the context of zoonoses (e.g., Da Silva et al., 2023; Keck and Lynteris, 2018; Lowe, 2010; Srinivasan et al., 2019); animal production (e.g., Blanchette, 2015; Wallace, 2016); companion animals (e.g., Gee et al., 2021; Han, 2022); or animal-assisted therapy (e.g., Franklin et al., 2007; Gorman, 2019; Sullivan et al., 2023). Another emerging strand has been oriented towards multispecies justice by addressing ethical and political implications vis-à-vis the health of all life-forms (e.g., Cañada et al., 2022; Celermajer and McKibbin, 2023). Such work examines whether and how conceptions of health can equitably encompass multiple lifeforms (human or not) that affect each other's health and well-being. This involves asking fundamental questions about what health entails; to echo Chao and Celermajer (2023, p. 6), who ask about justice^1^: “Who/what is *health* for and who benefits from it?”, “How ought we conceive of the subject of *health*?”, and crucially for this article: “What does *health* mean when refracted through a multispecies lens?”

In what follows, we discuss some key ways in which geographical and cognate literatures have engaged with the project of understanding and approaching health as a multispecies phenomenon. We mainly focus on the case of nonhuman animals to enable more in-depth and targeted inquiry, following an approach similar to Schuurman's (2024, p. 656) conceptualisation of ‘multispecies homescapes’ that analytically centres “human-animal relationality, while at the same time acknowledging that homes are created and experienced in a broad multispecies collaboration, also including plants and fungi.” To illustrate how these discussions might be of relevance to other life forms, and where useful for analytical purposes, we also provide select examples from scholarship that engages with non-animal life.

Through this critical review, we build on scholarly advances that critically describe the multispecies characteristics of health to investigate the possibilities for accounts of *multispecies health* that depart from exceptionalist notions of health that accord normative, i.e., ethical and political, primacy to the human. In doing this, we distinguish *multispecies health* from *health as a multispecies phenomenon* to signal the species-equitable character of the former. *Multispecies health,* to us, refers to concepts and practices of health that are equitable across human and nonhuman lifeforms.

Approaches to health beyond the human typically involve addressing animal or ecological health for human ends, or, less frequently, intervening for the health of the individual animal, like in the case of companion animals. These approaches, however, do not take into consideration the main factor (adversely) affecting the health of nonhuman lifeforms in contemporary societies: the structural and societal pursuit of human health and wellbeing, and that of a minority of valued nonhuman animals, as a part of developmental logics and practices (Narayanan, 2023; Srinivasan, 2022). Western medicine, for example, is built on an edifice of animals that are used for biomedical and veterinary innovation and safety testing. Another major arena through which human health is pursued is that of food and nutrition, which is also profoundly implicated in causing animal ill health and ecological destruction.

More broadly, key to fully understanding health is its co-constitutive relationship with ill health. The character of life on Earth is such that tensions between the health (and well-being) of various lifeforms, whether within or across species, are inevitable. Mainstream paradigms of health resolve these tensions by *a priori* privileging humankind, especially some humans (Ahmann, 2024; Sultana, 2012). By exploring the possibilities for multispecies health through the case of animals, we seek to illuminate ways of addressing these tensions that do not rest on situating nonhuman lifeforms as primarily means to human (health) ends.

Accounts of multispecies health that are equitable in how they address these tensions across lifeforms might seem inconceivable. But we take inspiration from how such tensions are addressed, with attention to equity, *within* humankind, such as when (human) population health is in tension with the health of (human) individuals. The ways in which this happens are imperfect, but for the most part, they rest on the normative principle that the pursuit of health for some humans should not cause ill health in other humans. It is this normative principle that translates as anti-anthropocentrism when it comes to multispecies health. What anti-anthropocentrism might entail in developing a paradigm for multispecies health is the core focus of the paper.

In what follows, we present a synthetic review of how geographical scholarship on animals has, through ontological and epistemological innovations, critically engaged with the human-centredness of conceptions and practices of health. We do not restrict our review to any one particular definition or understanding of health, but rather critically explore how health has been understood and approached through a multispecies lens in geographical literatures. Our review's approach, thus, is not unlike that adopted in Lorimer's (2012) review of geographical scholarship that goes beyond the idea of a singular and objective ‘Nature’.

While these scholarly developments have made significant advances in understanding the multispecies character of health, our analysis suggests that they have had only limited success in reconfiguring health in ways that dislodge the ethical and political primacy accorded to the human. We finally draw on wider multispecies justice scholarship to identify three avenues – specifically the concepts of *in-difference*, *intersubjectively affirmed agency*, and *re-animalisation* – that offer promise in terms of generating anti-anthropocentric accounts of multispecies health.

## Health as a Multispecies Phenomenon

More-than-human geographers, aiming to move away from human-centrism, have theorised nonhuman others as co-producers (and co-receivers) of health and ill health through their interactions with socio-economic and cultural settings (Hinchliffe, 2015, p. 32). A key feature of these literatures are epistemological and ontological innovations that have been deployed to investigate the multispecies dimensions of health.

### Epistemologies: from Separation to Connection

Geographical scholarship aiming to conceptualise health beyond the human has emphasised the need for epistemological innovation to overcome the centring of (privileged) humans as the main agents of health and as the “sole repositories of knowledge” about health (Lainé and Morand, 2020, p. 8). These literatures have advocated shifts in knowledge practices that recognise the interconnections between human and nonhuman lives, exceed disciplinary boundaries, and respect the plurality of health knowledges.

Some of this work has involved engagement with One Health (OH) and Planetary Health, approaches that have gained transnational prominence in policy and practice on health at the human-nonhuman interface. For instance, Marchese and Hovorka (2022, p. 5) highlight that OH “illuminates the inextricable and, indeed, unsustainable interconnections of animals, humans, and the broader ecosystems”. Similarly, Singer (2016, p. 352) flags Planetary Health's recognition that the “health and well-being of human communities also are multiply linked to the environment and to other species on Earth”. These literatures make visible particular human-nonhuman connections with impacts on health across species and advocate for intensifying dialogue across disciplines to better understand and intervene in these connections (see Geiger and Hovorka, 2015).

Others have identified a transdisciplinary dominance of epistemologies that replicate colonial structures in hindering understandings of health as multispecies and as cementing injustices, whether between species or intra-human (Malfrán and Baquero, 2023, p. 3). For instance, as Friese and Nuyts (2017) explain, OH tends to be operationalised through a hierarchical approach that advocates intersectoral collaboration for surveillance of and intervention in human-nonhuman interactions to improve and secure (some) humans’ health. A paradigmatic example of this can be found in the collaborations between the medical and veterinary sciences to keep farmed animals in the particular state of health needed for productivity and to prevent zoonoses (Van Patter et al., 2023, p. 3). Farming and pharmaceutical industries in the (transnational) Global North have typically been the main beneficiaries of this.

To others, such as Baquero et al. (2021, p. 9), epistemic injustice has been a key feature of contemporary approaches to health, such as Planetary Health, in how they “favor the interests of the global North, dictating what should be understood by health, how health problems should be solved, and how people should live to avoid them”. More generally, Gillespie et al. (2024) highlight how (mainstream) knowledge production about human-animal relations is shaped by what is considered legitimate in certain locations of academic esteem in the Global North. On the whole, this strand of inquiry asks questions concerning who the bearer of multispecies/interspecies health knowledge is, who the expert is, and who is legitimised to apply such knowledge and expertise – in short: who is the *Anthropos* (Probyn-Rapsey, 2018).

For example, writing about rabies prevention programmes within indigenous communities in Australia, Brookes et al. (2020, p. 8) argue that relying only on veterinary input is a “single-sided unsustainable approach to dog management which can undermine and detract within-community dog management”. In this sense, subverting dominant ways of knowing requires not only building connections between a diversity of disciplines, but also recognising a diversity of lived experiences and truth systems on how to live healthy lives with others – humans and otherwise – including those that might be experienced antagonistically (Langdridge and Flowers, 2013).

To address epistemic injustice, scholarship at the intersection of more-than-human geographies and health has aimed to understand peoples’ situated knowledges of interspecies health. For instance, Srinivasan et al. (2019) discuss the experiential, practical knowledge of people who live and work on the streets about safety around free-living dogs, arguing that rabies prevention programmes need to incorporate these insights. In a different context, Hinchliffe et al., (2021) explain how actively engaging with the perspectives of farmers vis-à-vis the political-economic conditions that shape their aquaculture practices offers fresh insights on how antimicrobial resistance can be effectively addressed.

A step further has involved epistemologically recentring the nonhuman by validating nonhuman lifeforms as knowledge producers. Scholars have explored whether and in what situations nonhuman animals are bearers of knowledge about their own health (Braverman, 2022, p. 8; Curtis, 2014). For instance, Lainé and Morand (2020, p. 6) have argued for an approach that “invites us not to choose between human or animal, but to carefully look at the network of relations they built in their shared environment”. This means not only looking at how local human medical know-how emerges, but also the knowledge possessed by animals about their own health. Referring to the work of Lainé (2018) in Laos, they suggest that, while caregiving for working elephants in Tai-Lue villages might be based on local ethnoveterinary practices, elephants’ knowledge is also key: elephants can “supplement them [their diets] if necessary thanks to the abundant diversity of the spaces they cross (…)” – “(…)the forest is the equivalent of a pharmacy for [them] (…)” (Lainé and Morand, 2020, p. 7). Elephants also usually prefer to be alone in the forest when they are sick, avoiding human contact.

Other animals similarly showcase knowledge about how to avoid unpleasant interactions with humans, protecting their health and safety, and often that of the humans they coexist with as well. For instance, in research on macaques in urban Singapore, Yeo and Neo (2010) discuss how some human residents seem to be aware of the strategies and knowledge macaques enact to avoid conflict with them. For example, Edward, a participant in their research, explained how macaques seemed

“(…) to be able to know our human's sense of territory, and will stay at a distance and observe me, at times seems scared of me. When I am around, they will keep a distance, like knowing that's my territory and it should not get there” (p. 693).

This is often the case with animals in non-urban environments too, who show human-averse behaviours and avoid human encounters (Jolly et al., 2022, p. 11; Knight, 2009). However, animal knowledge and practices about safeguarding their health can sometimes be perceived as negative from a human standpoint and therefore not identified as health knowledge at all. For instance, barking at people or vehicles could be considered as a canine strategy of health-preservation, but is one of the top sources of human complaints about street dogs (Srinivasan et al., 2019, p. 4). In effect, what health means is shaped by what is recognised knowledge about health and who is legitimised to bear it and act upon it.

### Relational Ontologies: Nonhuman Agency and the Co-Production of Health

The epistemological advances discussed in the previous section are based on ontological understandings of animals as agential subjects who bear health knowledge. This is different to considering them as passive creatures or objects of human knowledge and action. Expanding the epistemological basis of how health is understood has, therefore, been accompanied by the ontological recentring of nonhuman life and human-nonhuman relations in projects of health making and unmaking. This emphasis on ontology is shaped by the influence of posthumanism (Andrews, 2019), particularly Haraway's and Barad's work on knowledge production, which argues that “the knower and ‘things’ do not pre-exist their interactions but emerge through, and as part of, their material-discursive intrarelating” (Gherardi, 2022, p. 107).

This body of work argues that relational ontologies are crucial to understanding the multispecies character of health. As Anderson (2022, p. 269) explains, when thinking about risk and health, our perceptions

are still embedded in [a] dated animal ontology, rather than embracing a post-animal process ontology, which would expand the scope and quality of relationality. It may be a useful exercise to try to imagine the “animal” otherwise, as borderlands not boundary, as co-immune not immune, as interconnected and networked.

According to Keck and Lynteris (2018, p. 25) this imagining ‘the animal otherwise’ requires ontologically recognising animals as not just “passive carriers of long-term human symbols but as actors in unstable and vulnerable networks of coexistence”. For instance, Mitchell (2002) and Beisel (2017) explore how small animals like mosquitoes can shape human (health) projects, whether malaria eradication campaigns or dams. In a similar vein, Brown and Kelly (2014), in their work on viral haemorrhagic fevers, call for new approaches that pay attention to the “varied encounters that lead to transmission” and to animals as agentive co-participants, and not simply vectors of disease (p. 286).

Through allied perspectives, geographers and other social scientists have explored the capacity of nonhuman lifeforms to produce health or ill health through their entanglements with human society. In these literatures, the analysis of disease and health overcomes ontological dualisms to focus on the situated socio-ecological conditions that make certain relationships pathogenic or not. For example, transformations led by capitalist development can rapidly intensify the entanglements between humans, animals and pathogens, as seen in deforestation or urbanisation, with detrimental health outcomes (Wallace et al., 2020). In this view, health needs to be understood not through the lens of absence/presence of a nonhuman lifeform but through concepts such as relational folds and intensities (Hinchliffe et al., 2013), ecologies of practice (Mather and Marshall, 2011), assemblages (Andrews, 2019, p. 1113) and viral clouds (Lowe, 2010). As Wallace et al. (2020, pp. 6–7) point out, “epidemiologies have themselves turned relational, felt across time and space.”

The Harawayian idea of relationality emerging from inter-personal embodied experiences is an associated framework that has been used to conceptualise health at the human-nonhuman interface. As Greenhough (2011, p. 165) describes, for Haraway, “ontological politics involves the creation of novel body-environment ontologies, where medical conditions mark the starting point for developing new embodied ways of engaging with the world and other species within it”. Also centred on the body, Lorimer's (2017) discussion of hookworm-human relations rethinks the human as “a porous and ecological figure (…), whose life course microbial exposures come to configure identity, subjectivity and health outcomes” (p. 554). Beyond animals, the deployment of such ontologies can also be seen in Langdridge and Flowers (2013) analysis of the embodied experiences of gay men and their learning to become-with HIV.

Some of these literatures have argued that expanded “ontological sensitivities become a corporeal basis for renegotiating encounters with the world”, not as a basis for new privileges, but for greater response-abilities towards nonhuman others (Greenhough, 2011, p. 164). In other words, a different kind of (relational) ontology can be the basis of an ethics of relationality that decentres the human. However, how these relational onto-epistemologies might actually counter anthropocentrism in health frameworks (and beyond) remains debatable. We discuss this next by exploring their ethical and political implications.

## The Ethics and Politics of Multispecies Health

(…) [Annemarie Mol] demonstrates that any intervention in health and disease—biotechnological or otherwise—is best understood through an appreciation of how it is ‘situated’ within specific socio-material environments and practices of care (Greenhough, 2011, p. 160).

A major point of emphasis in the literatures reviewed above has been the ethical possibilities that emerge from interpersonal interactions between human and nonhuman agencies in health contexts. Such direct encounters allow for situated knowledge and caring practices that are responsive to specific multispecies contexts, and that go beyond the “fixed prioritisation of the health of specific life forms”, thus enabling multispecies health (Cañada et al., 2022, p. 22).

For example, Lorimer's (2020) work on ‘the probiotic turn’ shows how people's embodied relationships with and situated knowledge of organisms considered to be pathogenic (in mainstream conceptions of health) can lead to the re-evaluation of the healthiness of and the caring cultivation of these relationships. Hinchliffe and Ward (2014) similarly describe how the relational knowledge of industrial farmers enables a change from exclusionary visions of ‘pathogen management’ towards ‘immune-preparedness’ that has positive outcomes for pig and ecological health, and for farmers’ economies.

In a different geography, Porter's (2013, p. 145) ethnography of avian flu in Vietnam discusses the importance “of reflecting on and refashioning how to live with animals in a ‘One Health’ order”. Such reflections on health as “multispecies matters” (Gorman, 2021, p. 211) point to the potential of human–nonhuman embodied interactions in producing approaches to health that are more attentive to nonhumans. For example, in the context of food and agriculture, this could mean a shift away from industrial farming to higher-welfare systems that allow for better knowledge of and care for the health of the animals that are farmed (Marchese and Hovorka, 2022).

Related arguments can be found in debates on animal experimentation. Davies (2012), working on laboratory mice, proposes moving from universal ethical principles and focusing instead on ethics that derive from embodied human–animal entanglements. To Davies, this ethical approach enables “more complex understandings of animal lives” (p. 635). For example, Greenhough and Roe (2019), working with laboratory technicians’ accounts of their relationships and attunements with mice, show how both care and violence often become entangled in ways that escape rationality. While acknowledging that these mice's lives are shaped by structures of violence, they argue that direct encounters have the potential to change the everyday fabric of human–animal relationships (and therefore animal health) by recentring the nonhuman as subjects of moral worth.

Similarly, in Dam et al.'s (2020) analysis of piglets as infant models in biomedical research on neonatality, the laboratory

was a ‘space of encounter’ with nonhumans (…) and spurred an opening in thought that enabled the research team (…) to respond more carefully to piglets [who sometimes received] the same meticulous care as hospitalized infants (p. 8).

This situated mode of attention and knowledge means that tensions and conflicts between human and animal health, such as those seen in the laboratory, are often addressed through interactions that emerge at the micro-scale.

However, as Lynteris (2019, p. 2) notes in the context of zoonoses, while relational approaches

do contribute to a much-needed shift in the intellectual landscape as regards the impact of animals on human health, their practical and political limitations are revealed each time there is an actual epidemic crisis. Then, all talk of One Health, multispecies relationships and partnerships melts into thin air, and what is swiftly put in place, to protect humanity from zoonotic or vector-borne diseases, is an apparatus of culling, stamping out, disinfection, disinfestation, separation and eradication (…).

Building on this insight, we suggest that these practical and political limitations do not necessarily stem from failing to make relational frameworks work in moments of crisis, but are rather intrinsic to *how* animals and nonhumans have been onto-epistemologically centred in scholarship on multispecies health. For example, which and whose knowledges are legitimised do not necessarily translate to developing anti-anthropocentric approaches to health, which are equitable across species lines. In the examples discussed here and Section 2.1, health knowledges remain anthropocentric in their ethical and political prioritisation of different human interests, curiosity, and desires, even if there is an attempt to exceed established norms about what counts as valid knowledge.

A focus on encounters and relationality does have the potential to disrupt established ways of thinking about, knowing, and interacting with non-human animals. Yet, how these encounters and relationships are made sense of, as well as their outcomes, are always mediated by pre-existing human values, structures, and worldviews – which are predominantly anthropocentric. In other words, anthropocentrism implicitly shapes the limits and possibilities of most inter-personal encounters and care, and therefore, health in multispecies relationships.

A clear example of this is offered by Mc Loughlin (2024, p. 8), who explains how bringing farmed pigs closer to humans through slaughterhouse tours in Denmark did not lead to positive outcomes for these animals; such encounters were carefully curated to convince visitors that industrial slaughter was “legitimate, justifiable, and absolved of moral equivocation (…)”. Similarly, as Lorimer (2015) explains, encounters with animals in settings like zoos or eco-tourism take place within broader structures of animal commodification and a privileging of the experience of (and for) particular humans. This can also be seen in contexts such as ‘pet’ cafés (Robinson, 2019; Zhao et al., 2024) or animal-assisted therapy (Gorman, 2017; Taylor and Carter, 2020), which are often examined for their multispecies health possibilities, but where human values and objectives nonetheless prevail over animal health and wellbeing.

In essence, scholarly advances on the potential of relational ethics can overlook the extent to which concepts and practices of multispecies health remain affected by wider structures that are imbued with material and normative anthropocentrism. They, thus, tend to fall short of engaging with nonhuman lifeforms as equivalent “multispecies partners” in health (Deckha, 2022, p. 165). The latter requires not only re-centring animals as knowledgeable, active agents who might benefit from new ethical partnerships, but also recognising them as integral political subjects of their own health, even in situations when their health interests might oppose human health. Moving beyond such anthropocentrism, to Probyn-Rapsey (2018, p. 61), “needs multiple lenses, optics, but also mirrors, [and] a feeling for blind spots…” In the discussion that follows, we draw on this insight to explore possibilities for anti-anthropocentrism in accounts of multispecies health.

## Towards Anti-Anthropocentric Health Politics

Narayanan (2023, pp. 180–181), in a critical review of environmental geographies in this journal, argues that there is a fundamental difference between considering our entanglements with nonhuman others and the active process of becoming anti-anthropocentric.

In this paper, we have so far explored the different ways in which nonhuman animals are included in conceptualisations of health. These have primarily entailed epistemologically and ontologically recentring them in health knowledges and acknowledging their agency in projects of health. We have argued that while the concepts and approaches reviewed so far have provided crucial insights about health as a multispecies phenomenon, as Probyn-Rapsey (2018, p. 58) reminds us, anti-anthropocentrism is not a quality of all analyses that recentre animals ontologically and epistemologically.

What then, if any, is the way forward for equitable accounts of multispecies health that do not *a priori* rest on the ethical and political primacy of the human? We identify three avenues for advancing anti-anthropocentric frameworks for multispecies health by tweaking current approaches to onto-epistemological innovation through: a) an epistemic shift from purposively knowing animals to cultivated practices of ‘in-difference’; b) the ontological deployment of a more narrowly specified understanding of animal agency; c) a reorienting of ethical and political focus from animals to humans.

### Epistemology: In-Difference

The first avenue, in-difference, is what Narayanan (2023, 185) calls “a mindful human indifference to animal difference”, which, combined with attentiveness “to individuals, can serve as a basis for ‘progress’ as multispecies liberatory futures”. This attention to individuality and how it intersects with conceptualisations of animals as collectives has been explored in wider geographical scholarship (Bear, 2011; Buller, 2013; Srinivasan, 2014). However, practices of active in-difference to animals as a foundation for multispecies health remain underexplored; as discussed earlier, scholarly emphasis has been on increasing diversity in ways of knowing animals. In contrast to common understandings of ‘indifference’, here *in-difference* is understood not as a failure to recognise the Other (Hynes, 2016, p. 27) but as “mutually existing *in difference* rather than being different beings seeking to grasp, gaze, admire, and master the difference of others” (Davé, 2023, p. 6; our emphasis).

In-difference (in how nonhumans are known) is key for equitable cohabitation amongst different lifeforms, including those who may have characteristics that are antagonistic to, or of instrumental value to, human health. Such in-different cohabitation, in turn, is a precondition for multispecies health: health gains meaning only in the context of being able to live and pursue one's own telos – the “full range of possible matterings unique to different sorts of animals” – instead of being primarily a means (or obstacle) to another's end (Rollin, 2012, p. 76). As Greenhough, drawing from Canguilhem, notes, ill health lies in the prevention of “a living being from following their normal day-to-day routines; when their course of life is disrupted” (2023, p. 492).

For example, in the backdrop of transnational public health concern about rabies and bites, Srinivasan et al., (2019, p. 8) discuss how people's narratives about free-living dogs exemplify the kind of in-difference that allows (animal) difference to exist without “seeking to grasp, graze, admire, and master” it (Davé, 2023, p. 6):

[Street dogs] are integral inhabitants of the multispecies city, and like their human counterparts, can be involved in positive, negative and neutral interactions with their human and nonhuman cohabitants. They can be nuisances and pose health risks, but they are also vulnerable beings that are tolerated, cared for and cherished.

As one of their interview participants in Chennai puts it: “There would be about 100–200 dogs in this neighbourhood…Only if we bother them, they bother us… These dogs live along with people, like people (*makkaloda makkala vazhuthu*)” (p. 7).

Research in other regions, such as Goa by Corfmat et al. (2023), Azerbaijan by Ihar (2022), and Mexico by Ruiz-Izaguirre et al. (2020) corroborates this. The latter also explains how villagers who co-exist with *callejeros* (free-living dogs) “perceive (grownup) village dogs as adults. They are *capable of caring for themselves and others* (children, drunk men, domestic animals), capable of decision making, capable of asking for help when needed, and both *capable of and subject to judgment*” (p. 523; our emphasis).

These quotes present a picture of free-living dogs as independent animals that live amongst, and in varied (non) relationships with humans. These relationships exceed public and animal health conceptualisations and knowledge of these animals as carriers of disease and/or homeless pets, which subject them to killing, captivity, or neutering (Meyer et al., 2022). In these contexts, street dogs are able to pursue their telos – and health – because of ‘in-difference’ that unsettles the idea that closer relationships entail “positive mutuality and connection” (Ihar, 2022, p. 211). Rather, ‘in-difference’ manifests as the willingness to accept, or at least not disallow, animal difference, even in the context of potential mutual risk or harm.

Jolly et al. (2022, p. 9) identify a similar ethos amongst the forest-dwelling Kattunayakan tribe, who share home habitats with elephants, bears, and wild boars: community members expressed “consideration towards wild animals” – as opposed to active concern or care. Conejero et al. (2019, p. 69), similarly show how one of the main attitudes of many people living alongside wild pigs in Barcelona (Spain) is “respect” towards them (see also Arregui, 2023).

In all these cases, it is (human) in-difference that enables animals to pursue their own telos as free-living beings instead of being subsumed under human knowledge, which more often than not, produces regimes of entangled care and control that undermine nonhuman autonomy (see Giraud and Hollin, 2016) . This, in turn, opens up possibilities for multispecies health beyond what is imaginable under current human-directed health imaginaries

In the context of animals that are located entirely within human regimes, such as those that are farmed, in-difference would manifest as reconfiguring societal conditions so that their lives could potentially become indifferent to us, structurally re-allowing them to “have more important things to think about than merely being our negative foils” (Probyn-Rapsey, 2018, p. 51).

In both contexts, free-living and farmed, in-difference implies a shift from the current epistemological impetus, as outlined in Section 2.1, to fully know the animal Other for particular ends, whether for their own good, or for human good, or for the good of wider collectives. Rather, in-difference entails the ethos and practices of letting the Other remain in their (unknown) difference. In such a paradigm, ‘knowing’ the Other unfolds in ways that respect their autonomy and individuality within shared communities, and is more likely to be incidental as opposed to intentional. Such in-difference, we suggest, is a minimum condition necessary for multispecies health.

### Ontology: Intersubjectively Affirmed Agency

The second avenue, related to the concept of agency discussed in Section 2.2, is also tied to the idea of telos. It consists of a carefully specified understanding of (nonhuman) agency as more than just affecting others or having effects on health phenomena. Rather, it is

the expression or manifestation of a *subjective* existence, agency means affecting the world in ways that reflect a subject's desires or will […] Agency can, but need not manifest subjectivity with explicit intention or deliberation […] [but] agency depends on whether and how our subjective existence is taken up by others (Blattner et al., 2020, p. 4).

This, then, is an intersubjective account of agency that acknowledges the limits on agency that can derive from both conscious and structural hierarchies and discriminations (Blattner et al., 2020, p. 4), including those that stem from anthropocentrism. In this account, (nonhuman) agency is not merely an analytical category for understanding complex health phenomena such as zoonoses. Rather, it becomes meaningful only when it is taken seriously and affirmed (by human society), i.e., when nonhuman desires or will are not disallowed or negated (Blattner et al., 2020, p. 4). For example, in a human context, the phrase ‘I feel I have no agency’ indicates that agency is not merely about the capacity to act or to have an effect, but also about being able to influence the outcomes of one's engagements with and actions in the world.

Intersubjectively affirmed agency assumes significance in a context where nonhuman agencies are habitually restricted or inhibited by societal structures and artefacts. For instance, Wadiwel's (2016) analysis of fishing technologies argues that their development is rooted in the goal of defeating fish resistance to being caught, while Srinivasan's research (2024) on urban natures highlights how the agencies of free-living plants and animals are more often than not met with suppression.

Blattner et al. (2020) explore how intersubjectively affirmed agency relates to animal health in their research on animal sanctuaries and animal agency,

one cow repeatedly indicated her strong preference to be part of the upper cow herd, and was allowed to do this despite her vulnerable health status, whereas the usual practice at VINE [the name of the sanctuary] is to encourage frail cows to join the Commons (with its much less rugged terrain). (…) The negotiability of these decisions, not just the outcome, is an important dimension of agency—allowing individuals to be heard, acknowledged, responded to, taken seriously, and to have the possibility to change an outcome to their liking. (p. 8)

This is echoed by Donaldson and Kymlicka (2015, p. 67) who, exploring farmed animal sanctuaries, stress that interspecies flourishing in such settings is contingent on the recognition of

animal residents as full and equal members of the community, with a right to help shape the community. This is impossible if paternalistic decisions regarding safety, resources, or human convenience continuously limit animals’ freedom and agency—their ability to explore ways of living, and communicate to us what they want.

The sanctuaries described here serve as an example to understand how rethinking multispecies health in anti-anthropocentric directions requires recentring animals’ agencies – but in the specific sense of the capacity to shape their lives and relationships in all respects – and not merely within the limits imposed by mainstream human societies. Together, these literatures on the uptake as well as suppression of nonhuman agencies show how multispecies health is contingent on nonhuman agency that is intersubjectively affirmed by humans.

### Ethics and Politics: Re-Animalisation

The third avenue draws on the concept of ‘re-animalisation’. i.e., “resituating humanity as one among other animals, and relearning how to inhabit this world accordingly” (Srinivasan, 2022, p. 353). Srinivasan, in a departure from the scholarly emphasis on recentring *nonhumans* ontologically and epistemologically, argues for the redirection of ethical and political attention to “the *human* as a necessary response to the Anthropocene” (2022, p. 353). They highlight the ‘logics of protection-sacrifice’ that characterise contemporary approaches to human health and wellbeing, and which almost always harm and displace vulnerable humans and nonhumans while seeking to achieve particular visions of the ‘good’ human life. To them, therefore, addressing multispecies injustice therefore requires “re-animalisation”:

From a vision of a good human life premised upon insulation from the vulnerabilities inherent in living on this planet, we need to examine what it means to live as part of nature, as one among other animals. Equally crucial is a fundamental shift in approach to inequities. Instead of addressing social, ecological and animal injustices by ‘shoring up’ and seeking protections for vulnerable human or nonhuman Others, the focus would be on more equitably distributing the risks of living on this earth so that they are not borne primarily by marginal people and nature (p. 361).

With reference to multispecies health, the lens of re-animalisation suggests that multispecies health *does not entail* knowing, protecting or recentring animal health, but rethinking the meaning and weight accorded to human health in relation to that of other living beings. For instance, resituating humanity within nature implies asking, of humans, questions that are typically restricted to animals: what is the role of humans/particular humans, in the making of health and ill-health? How does the pursuit of human health affect the lives and ill-health of Others, whether nonhuman or human? Even beginning to contemplate humans as one among other animals, as challenging as it may be, upturns established approaches to health which more often than not are based on “the delay of human mortality and the reduction of human vulnerability to disease by deliberately creating ill health in, and hastening the deaths of, other animals” (Srinivasan, 2022, p. 355).

Re-animalisation as a pathway to multispecies health, therefore, focuses on unravelling the constitutive relationship human health has with nonhuman ill-health.

These three avenues have both empirical and conceptual relevance. Empirically, they offer analytical lenses with which to investigate health projects and phenomena by asking to what extent they contain elements of in-difference, intersubjectively affirmed animal agency, and/or re-animalisation; conceptually, they open up possibilities for experimenting with ideas and practices of health that escape and exceed the primacy of human health as it is currently understood.

## Conclusions

This article has critically examined what it means to situate nonhuman animals more equitably in understandings of health. We have done so by mapping how geographical literatures on health as a multispecies phenomenon have built on onto-epistemological innovations on human-animal relationships. This encompasses, amongst others: (1) expanding understandings of health knowledge *about* and *by* nonhumans, and (2) rethinking the ways in which nonhuman agency and more-than-human relationality might reinforce or disrupt ideas and interventions on health.

The article explains how this onto-epistemological scholarship has foregrounded paradigms of more-than-human relationality and their ethical potential in enabling non-anthropocentric accounts of health: embodied interactions allow for closer knowledge of and care for nonhuman health. However, we argue that such interactions and relationalities are almost always situated within the ethical and political coordinates of human-centred worlds. Critical scholarship can centre human interests, values, and wellbeing in health making and unmaking, even while centring nonhuman animals and more-than-human relationality onto-epistemologically.

Therefore, when it comes to health, the ‘residual anthropocentrism’ (Lulka, 2009) we find in such approaches does not allow the redressing of how – in similar terms to Ahmann (2024, pp. xi–xii), who writes with reference to humans in Baltimore – some nonhuman

lives end sooner so as to enable futures elsewhere (…) You might register the deaths, but not the fundamental reasons for them, nor the steely logics that make them reasonable. Not the quiet, long-term forces that bind these foreclosed futures to the stable lives and secure worlds of privileged others.

These underlying steely logics, while reducing the vulnerability of (some) humans by “displac[ing] and exploit[ing] both nonhuman and subaltern human life”, render *reasonable* current configurations of who and what matters in health and constitute the politics of anthropocentrism in health (Srinivasan and Kasturirangan, 2016, p. 128). The danger our article raises concerns about is that, in trying to challenge these ‘steely logics’, we inadvertently reinsert nonhuman Others and their knowledges and agencies, deeper into human-exceptionalist frameworks of health and wellbeing. As such, these efforts could inadvertently reinforce these very same “stable lives and secure worlds of privileged others” (Ahmann, 2024, p. xii).

To overcome this residual anthropocentrism, in the article, we identify three incipient conceptual pathways towards anti-anthropocentric accounts of multispecies health: *in-difference*, *intersubjectively affirmed agency*, and *re-animalisation*. In common with all of these is the decentring of the (privileged) human as the centre of gravity of health. These tools also help to avoid the tendency to amplify the focus on the nonhuman through new onto-epistemological lenses, without fully addressing the ethical and political oversights that lead to even multispecies accounts of health being recast as renewed injustices for nonhuman lifeforms.

Specifically, we see: (1) in-difference as an active *epistemological* (but not ethical) disengagement that can avoid reinforcing and centring more-than-human knowledge in contexts where these might be structurally exploiting the health of nonhumans; (2) intersubjectively affirmed agency as an *ontological* framework that asks to what extent nonhuman agencies are enabled or disallowed by human society, with knock-on health impacts; and finally, (3) re-animalisation as a *politics* of the more-than-human that avoids exceptionalising human wellbeing as the ultimate goal of health, and that includes the possibility of countering the ‘unquestionability’ of human futures that can only be sustained on the ill-health of nonhuman lifeforms.

To conclude, we suggest that the conceptual avenues we have charted offer reflective tools for (a) understanding health beyond the human and for recognising nonhumans’ health and needs as important on their own, yet (b) still be attentive to how efforts to do so might be limited – especially by how nonhuman (ill) health is deeply embedded within human systems and worlds. At times, taking nonhuman animals’ health seriously might mean, instead, to redirect analytical attention towards approaches to human health and their structural connections to the ill-health of animals. Multispecies health, then, is less about knowing, caring for and improving nonhuman health, and more about undoing the systemic normative and material drivers of nonhuman ill health.

## Notes

1. The original quotations refer to justice, not health.
